# Assessing the outcomes of participatory research: protocol for identifying, selecting, appraising and synthesizing the literature for realist review

**DOI:** 10.1186/1748-5908-6-24

**Published:** 2011-03-20

**Authors:** Justin Jagosh, Pierre Pluye, Ann C Macaulay, Jon Salsberg, Jim Henderson, Erin Sirett, Paula L Bush, Robbyn Seller, Geoff Wong, Trish Greenhalgh, Margaret Cargo, Carol P Herbert, Sarena D Seifer, Lawrence W Green

**Affiliations:** 1 Department of Family Medicine, McGill University, Montréal, Canada; 2 Research Department of Primary Care and Population Health, UCL, UK; 3 Centre for Health Sciences, Barts, and The London School of Medicine and Dentistry, London, UK; 4 School of Public Health, University of South Australia, Adelaide, Australia; 5 Schulich School of Medicine & Dentistry, University of Western Ontario, London, Canada; 6 Community-Campus Partnerships for Health, Seattle, USA; 7 Department of Epidemiology and Biostatistics, University of California at San Francisco, San Francisco, USA

## Abstract

**Background:**

Participatory Research (PR) entails the co-governance of research by academic researchers and end-users. End-users are those who are affected by issues under study (*e.g.*, community groups or populations affected by illness), or those positioned to act on the knowledge generated by research (*e.g.*, clinicians, community leaders, health managers, patients, and policy makers). Systematic reviews assessing the generalizable benefits of PR must address: the diversity of research topics, methods, and intervention designs that involve a PR approach; varying degrees of end-user involvement in research co-governance, both within and between projects; and the complexity of outcomes arising from long-term partnerships.

**Methods:**

We addressed the above mentioned challenges by adapting realist review methodology to PR assessment, specifically by developing inductively-driven identification, selection, appraisal, and synthesis procedures. This approach allowed us to address the non-uniformity and complexity of the PR literature. Each stage of the review involved two independent reviewers and followed a reproducible, systematic coding and retention procedure. Retained studies were completed participatory health interventions, demonstrated high levels of participation by non-academic stakeholders (*i.e.*, excluding studies in which end-users were not involved in co-governing throughout the stages of research) and contained detailed descriptions of the participatory process and context. Retained sets are being mapped and analyzed using realist review methods.

**Results:**

The librarian-guided search string yielded 7,167 citations. A total of 594 citations were retained after the identification process. Eighty-three papers remained after selection. Principle Investigators (PIs) were contacted to solicit all companion papers. Twenty-three sets of papers (23 PR studies), comprising 276 publications, passed appraisal and are being synthesized using realist review methods.

**Discussion:**

The systematic and stage-based procedure addressed challenges to PR assessment and generated our robust understanding of complex and heterogeneous PR practices. To date, realist reviews have focussed on evaluations of relatively uniform interventions. In contrast our PR search yielded a wide diversity of partnerships and research topics. We therefore developed tools to achieve conceptual clarity on the PR field, as a beneficial precursor to our theoretically-driven synthesis using realist methods. Findings from the ongoing review will be provided in forthcoming publications.

## Background

Participatory Research (PR) is the collaborative co-governance of research, involving researchers and those affected by issues under study or who are in positions to act on the knowledge generated by research (*e.g.*, end-users including participants of an intervention, clinicians, health managers, policy makers) [1]. PR proponents claim that this approach enhances health outcomes by increasing cultural and logistical relevance of programs to their settings [1,2], promotes community empowerment [3], and facilitates the translation of research-generated health knowledge into practice [1,2,4-7]. Also suggested is that co-governance with end-users can unearth the social, political, and economic contexts that underpin both facilitators and barriers to knowledge and resources needed for health [1,8,9].

Despite its lauded benefits, there is a dearth of primary research and systematic reviews assessing the impact of PR on research and health outcomes. Assessment difficulties have been attributed to: the diversity of research methodologies, settings, and groups; the lack of standardized evaluation and reporting frameworks; and insufficient numbers of completed studies using a PR approach [10,11]. We addressed these challenges by conceptualizing PR assessment using realist review methodology [12], and specifically by developing a unique set of identification, selection, appraisal, and synthesis procedures to address the non-uniformity and complexity of the PR literature. By developing and applying these tools iteratively, we only retained studies that: were completed participatory health interventions; demonstrated high levels of participation by non-academic stakeholders (*i.e.*, excluding studies in which end-users were not involved in co-governing throughout the stages of research); and contained detailed descriptions of the participatory process and context, required for realist synthesis.

Our rationale for applying a realist approach to this topic is described elsewhere [13]. Described here are the tools and procedures we developed and used for identification, selection, appraisal, and synthesis. Publication of our findings is forthcoming.

### Who we are

This systematic review is funded by a grant from the Canadian Institutes for Health Research, and is coordinated through the centre for PR at McGill (PRAM). PRAM's mandate is to: promote critical scholarship in PR; develop a multidisciplinary network of researchers; collaborate with funders and ethics boards to promote the development of PR guidelines; and support the competent use of PR through seminars, continuing medical education, faculty development workshops, consultations, resource development, and student training.

The research team consists of the PRAM core group of eight co-investigators and researchers (ACM, ES, JH, JJ, JS, PLB, PP & RS), five co-investigators from other institutions with expertise in PR or in realist review (CH, GW, LG, MC & TG), and seven knowledge user (decision maker) partners (see Appendix 1: Tables 1, 2 and 3). The partners were invited to participate to maximize relevancy and uptake of the review findings. They are administrators representing Canadian federal and provincial health research funding agencies and public health agencies, as well as an institutional ethics review board, and an organization for community-university engagement. The partners helped in shaping the initial review questions, writing or reviewing the grant proposal, providing feedback on the tools, and reviewing publication drafts. Guiding principles for the partnership were written at the start of the review (see Appendix 2). The core group met regularly during the research process to review progress, develop procedures, troubleshoot and maintain correspondence with the full team.

### Research Questions

Through the initial funding application process, the research questions were developed by the core group and sent to the partners to further define the aim of the proposed review according to their experiences and the priorities of their organizations. Consensus on the need to produce a comprehensive account of the benefits of PR and on the three review questions was reached.

Three research questions were:

1. What benefits, if any, can be observed from the collaborative steering of health intervention research by researchers and those affected by the issues under study or who would apply research results?

2. How can the benefits of such PR collaboration be conceptualized?

3. How do variations in program context and mechanisms influence the process and outcomes of collaborative health intervention research?

### Literature Search

A librarian-guided literature search was conducted in February 2009. The initial search strategy (see Appendix 3: Table 4) captured PR in all disciplines, including agriculture, education, health, management, and social sciences. This comprehensive search was built for two main reasons: publications pertaining to complex health-related PR interventions are located in academic journals in many disciplines (*e.g.*, social work), and retrieving conceptual frameworks and theoretical models of PR outside health disciplines was thought to be helpful in addressing our second research question. Synonyms and related terms were used such as community-based PR, action research, participatory action research, participative evaluation, and emancipatory evaluation. A total of 7,167 citations were retrieved.

### Tool development and coding procedure for identification, selection, and appraisal

Three tools were developed (for identification, selection, and appraisal) in March, June, and October 2009, respectively. Modifications were made during each stage after piloting. Each stage processed a different type of data: citations in identification; full-text papers in selection; and sets of publications in appraisal. Each criterion in the tools was coded '1' for yes, '0' for no, or '2' for unsure. Two reviewers independently coded all citations. A citation, a full-text paper, or a set of papers was retained if both reviewers coded '1' to all criteria in the tool. A third team member reviewed papers in instances of disagreement between the coders, and after discussion and debate within the team, cast a tie-breaking code.

### Construction of a WIKI page

A collaborative, private online workspace, called Wiki [14], was created to allow all members of the team to share knowledge, access information, comment, and interact via asynchronous discussion. Significantly, the wiki afforded co-investigators and decision-maker partners continuous access to the work in-progress. Members routinely added their work to the wiki at each stage of the review. All citations, full-text papers, excel sheets, meeting minutes, and other resources are stored on the website and available for all team members to access at any time.

### Identification

The identification tool consisted of three questions. This step funnelled the number of citations from 7,167 to 594.

### Identification Tool

1. Does the citation indicate health-related research?

2. Does the citation indicate PR?

3. Does the citation indicate some form of data (process or outcome)?

### Selection

The librarian (JH) retrieved the 594 full-text papers, which were read by two independent reviewers, using a selection tool initially comprised of six questions in June 2009, with an additional two questions added in October 2009. The first six questions were:

### Selection Tool

1. Does the full-text paper still indicate health-related research?

2. Does the full-text paper indicate that participation occurred in the following three areas:

a. partners were involved in identifying or setting the research questions?

b. partners were involved in setting the methodology or collecting data or analysing the data?

c. partners were involved in uptake or dissemination of the research findings (this requirement was loosely applied after consulting our co-investigators, because it was felt publication often predates uptake and the participatory effort in dissemination is often not addressed)?

3. Does the full-text paper describe the research setting? (indicate community-based, organizational, or other (describe))

4. Does the full-text paper indicate empirical research (*i.e.*, that there is some description of methods, data collection and analysis)? (Specify the methodology)

5. Does the full-text paper describe PR-related outcomes?

6. Does the full-text paper describe PR processes or contexts (or is there a reference to the process/context in a cited companion paper)?

Two hundred articles remained from 594 after filtering them through the selection tool. Due to the complexity of the dataset, we decided at this stage to further limit the scope of our review to community-based settings, and to participatory interventions. Our rationale was that: PR in all forms (community-based PR, organizational PR, action research) was too diverse to be assessed within one review; the complexity of PR benefits from community-based research provided a manageable set of studies; intervention research demonstrated more complexity of outcomes than non-intervention research, and would be best suited for analysis using realist review methods; and the pool of studies needed to be reduced to a manageable size for an in depth realist synthesis (analysis). Adding two questions reduced the pool to 83 studies. These were: Does the full-text paper indicate intervention research? Does the full-text paper indicate a community-based setting?

### Confirmation from principal investigators

Contact with principal investigators of all full-text papers retained after selection was undertaken because descriptions of programs, methods, and findings of PR interventions were found to be commonly described across a number of publications pertaining to the same intervention. It was thus necessary to confirm that we had complete sets of papers in order to fairly appraise projects according to the realist review approach. Before contacting authors, we read the article retained in selection to note if other companion papers were included in the list of references. For each study, we then sent our list of papers to the corresponding author or PI, and asked them to confirm that we had the complete set, or to send us additional documents. Eighty-three letters were sent via email and we received responses from 32 PIs (39%), either confirming that we had the complete set, or sending us additional publications. Only those sets of studies in which the contacted researcher responded to our request were retained for appraisal.

### Appraisal

The appraisal tool consisted of three questions. An additional 11 sets were eliminated after screening with the tool below, which left a total of 23 sets, comprising 276 documents that were retained for synthesis. See Appendix 4: Table 5 for a complete breakdown of the number of cases retained at each stage.

### Appraisal Tool

1. Did we receive an answer from the principle investigator confirming we have the complete set of publications for each study or providing additional publications?

2. Does the set of papers describe the outcomes in sufficient detail?

3. Does the set of papers describe the participatory process and context in sufficient detail?

For questions two and three, we deemed a set of publications to have sufficient detail if we were able to see at least one example of co-governance having an impact on the research processes or outcomes (*i.e.*, being able to create at least one CMO configuration).

### Synthesis

Background on realist synthesis methods can be found elsewhere [12]. Appendix 5 provides definitions of key realist review concepts: middle-range theory, demi-regularities, and context-mechanism-outcome configurations. The synthesis process is being undertaken in eight iterative and overlapping steps:

1. Searching for explanatory middle-range theories.

2. Preliminary annotating and extracting of data that pertain to PR processes.

3. Identifying demi-regularities based on annotated data.

4. Embedding context-mechanism-outcome configurations in the larger chronology of partnership events.

5. Sorting CMO configurations according to demi-regularities.

6. Refining CMO configurations, with particular attention to identifying the mechanisms;

7. Confirming or modifying our understanding of the demi-regularities based on refined CMOs.

8. Confirming the relevance of our identified middle-range theories as applied to these CMO configurations.

All steps in the synthesis were conducted by one of three members of the team (JJ, PLB, ES) and then cross-checked by one of six members (JJ, PLB, ES, ACM, JS, PP). Once we received confirmation from the PI that we had the complete set of papers for a given partnership, data pertaining to any effect of participation was annotated and extracted. That evidence was examined by the wider team in order to identify predictable patterns of behavior (demi-regularities) to explain typical outcome patterns. Due to the fact that project descriptions were written across multiple papers, we then mapped the project lifecycles using PREZI software. The lifecycles describe the chain of program activities, implementation steps, and descriptions of stakeholders. All data pertaining to the effect of the participatory approach, (which was annotated from the previous step), was incorporated in the map and configured in terms of the context, mechanism and outcome involved. For cross-checking by a second team member, these CMOs were referenced with the article, page, and paragraph number. All core team members confirmed the accuracy of the maps. The CMO configurations from the maps are currently being organized according to demi-regularity and being refined in terms of the mechanism. The final stages of our synthesis will include confirming or refining our understanding of the demi-regularities at play and the middle-range theories that provide an explanatory framework for how, why and in what circumstances PR works.

## Discussion

Published examples of realist reviews to-date have focused on evaluating sets of relatively uniform interventions or programs in terms of study topic, purpose and activities, (*e.g. *school-based nutritional programs [15] and web-based continuing education [16]). In contrast, our inquiry uses realist methods to assess a heterogeneous pool of studies that used a PR approach, regardless of study topic. This heterogeneity requires us to use a method that can make sense of the context and process data, which often takes the form of project descriptions and researcher narratives. In using realist methodology, we are able to assess the implementation chain in PR, and develop theoretical frameworks for linking PR processes with intermediate and final goals of research. Given the vast PR literature, we reduced the pool of studies in a systematic way to achieve an adequate level of uniformity of the retained set for synthesis. Disagreements in coding the literature led to valuable team discussions that clarified our position about what is, and is not PR. Protocol development was an inductive and iterative process in which we developed tools to assess the literature and made modifications as we progressed to enhance their fit with our field of investigation and our resource constraints.

Only 39% of PIs contacted responded to our request to confirm that we had their complete set of publications. We considered re-contacting authors at a later date to ensure they did not miss our first email attempt. However, despite this low response rate, we received an adequate sample size for our synthesis once companion papers were added. Our final number of sets (n = 23) is appropriate to the qualitative nature of realist review.

Although we had initially expected to be able to synthesize data across the diversity of the PR literature, we later saw the value in focusing solely on community-based health interventions as our area of investigation. Our decision was partly due to our limited resources, and partly to the fact that assessing all forms of collaboration in one review involved too many layers of complexity. Subsequent research may focus on other forms of collaboration, such as practice-based networks and action research in organizational settings.

Discrepancies in the two-person coding process and arbitration by a third party were learning opportunities in which we deepened our understanding and attained conceptual clarity on the heterogeneous PR practices found in the literature. The results of our systematic review process have led to a more comprehensive understanding of this diversity. We argue that this systematic process was needed to clarify how we conceptualize PR as we search for middle-range theories that explained our CMO configurations.

Our comprehensive literature search, the reproducible 'identification-selection-appraisal' process, and having two independent reviewers at all stages of this coding process constitutes a departure from review approaches using realist logic [12]. However, the process of implementing this rigorous and reproducible strategy ensured that we captured and filtered only relevant PR literature and expanded our understanding of the content area through debate and discussion of various cases during the search and retention of relevant papers.

## Conclusion

The tools we developed and used in this review enabled us to overcome barriers to PR assessment, and reduced the pool of studies to a manageable size, suitable for a realist synthesis of complex health PR. Reporting our identification, selection, appraisal and synthesis protocol here is a step in establishing transparency and replicability of our review process, and will facilitate the critical examination and dissemination of our review findings.

## Competing interests

The authors declare that they have no competing interests.

## Authors' contributions

All authors agreed on the need for a protocol paper. JJ drafted the paper with the assistance of ES. All other authors reviewed the manuscript and provided extensive feedback. All authors have read and approved the final manuscript.

## Appendices

### Appendix 1: List of partners (Tables 1, 2 and 3)

**Table 1:** PRAM Core Researchers

PRAM core group of researchers	Organization	Role
Ann C Macaulay	McGill Family Medicine	Principal Investigator

Pierre Pluye	McGill Family Medicine	Co-Investigator

Jon Salsberg	McGill Family Medicine	Co-Investigator

Jim Henderson	McGill Life Sciences Library	Co-Investigator

Robbyn Seller	McGill Family Medicine	Co-Investigator

Justin Jagosh*	McGill Family Medicine	Post-doctoral fellow

Paula L. Bush	McGill Family Medicine	Research Assistant

Erin Sirett	McGill Family Medicine	Research Assistant

**Table 2:** Out-of-town co-investigators

Out-of-town co-investigators	Organization	Role
Geoff Wong	University College London, UK	Co-Investigator

Trisha Greenhalgh	University College London, UK	Co-Investigator

Carol P. Herbert	University of Western Ontario, Canada	Co-Investigator

Margaret Cargo	University of South Australia	Co-Investigator

Lawrence W. Green	University of California at San Fransisco, USA	Collaborator

**Table 3:** Knowledge-user partners

Knowledge-users partners	Organization	Role
Susan Law/David Clements	Canadian Health Services Research Foundation	Federal Funding Agency

Marielle Gascon-Barré 2008-2009	Fonds de la Recherche en Santé Quebec	Provincial Funding agency

David L Mowat	Peel Region Public Health	Regional Public Health

Sylvie Stachenko/Sylvie Desjardins	Public Health Agency of Canada	Federal Public Health

Ilde Lepore	McGill Medicine Faculty Institutional Review Board	Institutional Review Board

Sarena D. Seifer	Community-Campus Partnerships for Health	Community-Engaged Scholarship

* Corresponding Author

### Appendix 2. Principles guiding our collaborative process with partners and decision-makers

1. The core members of the PRAM review team will maintain regular contact with all reviewers and partners for advice and guidance throughout the stages of the review.

2. Reviewers and partners will have open access to all documents, tools, and reports from the WIKI site.

3. Reviewers and partners will all be invited to participate in the coding, selection, and analysis processes.

4. Notifications will be sent out when new and important updates are posted on the WIKI, (*e.g.*, after each monthly meeting), and asking for feedback when needed.

### Appendix 3: Table 4

**Table 4:** Details of Search Strategy

SEARCH STRATEGY KEY WORDS	DATABASES
Initial search strategy: 'Participatory research' OR 'Participative research' OR 'Action research' OR 'Collaborative inquiry' OR 'Participatory rural appraisal' OR 'Participatory appraisal' OR 'Emancipatory research' OR 'Social reconnaissance' OR 'Empowerment evaluation' OR ((participatory[title] AND (research[title] OR design[title])) OR 'participatory research'[title] OR 'action research'[title] OR (('Consumer participation'[mesh] OR 'Consumer advocacy'[mesh] OR 'Community-institutional relations'[mesh]) AND (research[title] OR research[mesh])) OR 'dialectical research' OR 'conscientizing research' OR 'participatory learning research' OR CBPR OR 'community-based participatory research' OR 'community based participatory research' OR 'community based action research' OR 'participatory evaluation' OR 'participative evaluation' OR 'community-driven research' OR 'community-driven action research' OR 'action science' OR 'community-partnered action research' OR 'cooperative inquiry' OR 'dialectical inquiry' OR 'appreciative inquiry' OR 'decolonizing methodologies' OR 'democratic evaluation'OR 'recherche participative' OR 'recherche action' OR 'recherche collaborative' Final focused strategy: 'Participatory research' or 'Participative research' or 'Action research' or 'Collaborative inquiry' or 'Participatory rural' or 'Participatory appraisal' or 'Emancipatory research' or 'Social reconnaissance' or 'Empowerment evaluation' or cbpr or 'community-based participatory research'	Focused strategy used: • Ovid Medline (international biomedical journal literature) • EMBASE (international biomedical literature) • PsycInfo (international biomedical literature) [which included numerous citations from Proquest Dissertations] • ISI Web of Knowledge (journal literature of all disciplines; citation searching) Additional sources used in initial searching only; results not included in screening: • PubMed • CINAHL (Cumulative Index to Nursing and Allied Health Literature; international) • Cochrane Library (databases of systematic reviews and of clinical trials) • ERIC (journal articles, reports, and other types of publications in education) • Social Work Abstracts (international social work literature) • Scopus (journal literature of all scientific disciplines; citation searching) • Proquest Dissertations and Theses • Additional sources for report/'grey' literature: Internet via Google and Google Scholar; New York Academy of Medicine Grey Literature Report and other sources listed in GreyNet

### Appendix 4

**Table 5:** Identification, selection, and appraisal flow chart

Identification	Identification tool:
7167 citations retrieved from the literature using broad search strategy (Appendix 3) and scrutinized using identification tool	1. Does the citation indicate health-related research? 2. Does the citation indicate PR? 3. Does the citation indicate some form of data (process or outcome)?
Selection	Selection tool:
594 citations and their full-text articles examined using selection tool	1. Does the full-text paper still indicate health-related research?
	2. Does the full-text paper indicate that participation occurred in the following three areas:
	a. partners were involved in identifying or setting the research questions?
	b. partners were involved in setting the methodology or collecting data or analysing the data?
	c. partners were involved in uptake or dissemination of the research findings?
	3. Does the full-text paper describe the research setting? (indicate community-based, organizational, or other (describe))
	4. Does the full-text paper indicate empirical research (*i.e.*, that there is some description of methods, data collection and analysis)? (Specify the methodology)
	5. Does the full-text paper describe PR-related outcomes?
	6. Does the full-text paper describe PR processes or contexts (or is there a reference to the process/context in a cited companion paper)?
	7. Does the full-text paper indicate intervention research?
	8. Does the full-text paper indicate a community-based setting?

Appraisal: 83 sets of articles assessed using appraisal tool (contacted PIs to ensure all publications for each study)	Appraisal tool: 1. Did we receive an answer from the principle investigator confirming we have the complete set of publications for each study or providing additional publications?; 2. Are there rich descriptors of the outcomes?; 3. Are there rich descriptors of the participatory process?

23 studies comprising 276 publications for qualitative synthesis using realist review methods	

### Appendix 5

#### Definition and use of realist review concepts

Three main concepts described here are derived from Pawson et al's text on realist review methodology [12]. These are: middle-range theory; context-mechanisms-outcome configurations; and demi-regularities.

#### Middle-range theory

Pawson asserts that programs and interventions can be understood as theories put into practice. For a given intervention, the program theory(ies) may be intentional and explicit, or subconscious and implicit. Realist reviewers explicate these programme theories and study how they materialize in interventions to unearth causal explanations about their success and failure in differing circumstances. A program theory is considered middle-range when it is capable of retaining its relevance across multiple cases and in differing contexts. It is thus theory that is not abstract to the point of being disconnected from the actual workings of program implementation, yet, not specific to the point of being relevant to only one case.

#### Context-Mechanism-Outcome (C-M-O) configurations

CMO configuring is a heuristic used to generate causative explanations pertaining to the evidence. The process consists of aligning the context, mechanism, and outcome of a particular point of investigation to uncover the array of factors that may contribute to process or end results. A CMO may pertain to the whole program, or to certain aspects, and as such, one CMO may be embedded within another other. CMOs may also be configured in a series, in which the outcome of one CMO becomes the context for the next. Configuring CMOs is a step toward generating or refining the theory or theories that become the final product of the review.

#### Context

Context pertains to the 'backdrop' of programs and research. In our work for example, it pertains to the conditions connected to the development of research partnerships. This can include cultural norms and history of the community in which a program is implemented, the nature and scope of pre-existing social networks or previously built program infrastructure. It can also include geographic location effects, funding sources, opportunities, or constraints. Context can also be understood as anything that can trigger and/or modifies the behavior of a mechanism.

#### Mechanism

Mechanism pertains to what 'turns on' in the minds of program participants and stakeholders that make them want to (for example) participate or invest in programs. They may be cognitive or emotional responses, typically in relation to program resources being offered. Mechanisms are not synonymous to program strategies, which are intentional measures taken by program implementers. Identifying mechanisms advances the synthesis beyond describing 'what happened' to theorizing 'why it happened, for whom, and under what circumstances' based on participant reasoning or reaction.

#### Outcomes

Outcomes are either intended or unexpected, and defined as either intermediate or final. Examples of PR outcomes include increased levels of empowerment, education, knowledge, or development of program infrastructure and enhanced research processes. Examples of intervention outcomes include improved health outcomes, increased uptake of health services and enhanced research results.

#### Demi-regularity

'Demi-regularity,' originating from Lawson (1997) [17], suggests that human choice or agency manifests in a semi-predictable manner - 'semi' because variations in reoccurring, predictable patterns of behaviour can be attributed to differences in the contextual dimension from one setting to another [12]. For our review, we created a series of demi-regularities pertaining to the benefits of participation.
